# Simultaneous Enhancement of iron Deficiency Tolerance and Iron Accumulation in Rice by Combining the Knockdown of *OsHRZ* Ubiquitin Ligases with the Introduction of Engineered Ferric-chelate Reductase

**DOI:** 10.1186/s12284-022-00598-w

**Published:** 2022-10-31

**Authors:** Takanori Kobayashi, Keisuke Maeda, Yutaro Suzuki, Naoko K. Nishizawa

**Affiliations:** Research Institute for Bioresources and Biotechnology, Ishikawa Prefectural University, 1-308 Suematsu, Nonoichi, Ishikawa 921-8836 Japan

**Keywords:** ferric-chelate reductase, fortification, iron deficiency tolerance, *Oryza sativa*, ubiquitin ligase

## Abstract

Iron is an essential micronutrient for living organisms, but its solubility is extremely low under alkaline conditions. Plants often suffer from iron deficiency chlorosis in calcareous soils, which consist of approximately 30% of the world’s cultivated area, severely limiting plant productivity. Iron deficiency anemia is also a widespread problem in humans, especially in Asian and African people who take up iron mainly from staple foods containing low iron concentrations. Transgenic manipulation of genes involved in plant iron uptake, translocation, and storage has made improvements in enhancing iron deficiency tolerance or iron accumulation in edible parts, but these two properties have been characterized separately. We previously produced transgenic rice lines, with concomitant improvement of iron deficiency tolerance and grain iron accumulation by knocking-down *OsHRZ* ubiquitin ligases, which negatively regulate iron deficiency response and iron accumulation in rice. In the present report, we aimed to further improve the iron deficiency tolerance and grain iron accumulation of *OsHRZ* knockdown rice by the simultaneous introduction of the engineered ferric-chelate reductase gene *Refre1/372* under the control of the *OsIRT1* promoter for further enhancement of iron uptake. We obtained several transgenic rice lines with repressed *OsHRZ* expression and induced *Refre1/372* expression. These lines showed a variable degree of iron deficiency tolerance in calcareous soils, with increased iron accumulation in brown seeds under both iron-deficient and iron-sufficient soil cultures. Selected *OsHRZ* knockdown plus *Refre1/372* lines showed similar or better growth compared with that of singly introduced *OsHRZ* knockdown or *Refre1/372* lines in calcareous soils under both non-submerged and submerged conditions. After submerged calcareous soil cultivation, these *OsHRZ* knockdown plus *Refre1/372* lines accumulated 2.5–4.3 times and 17–23 times more iron concentrations than that of non-transformants in brown rice and straw, respectively, which was comparable or superior to a single *OsHRZ* knockdown line. Our results indicate that the combined introduction of *OsHRZ* knockdown and *OsIRT1* promoter-*Refre1/372* is highly effective in further improving the iron deficiency tolerance without compromising the iron accumulation of the *OsHRZ* knockdown effects.

**Supplementary Information:**

The online version contains supplementary material available at 10.1186/s12284-022-00598-w.

## Introduction

Iron (Fe) is an essential micronutrient for most organisms, including all plants and animals. Fe mediates various vital processes, including electron transfer for respiration and photosynthesis; numerous redox reactions and metabolisms, including DNA synthesis; and oxygen transport in the animal body, either as a constituent of heme, Fe-sulfur cluster, or as a ferrous ion (Fe^2+^) (Marshner 1995; Briat et al. 2015). Although Fe is abundant in the earth’s crust, its solubility is strictly limited, depending on pH and redox potential. In aerated soils, with a high pH, such as calcareous soils, which cover approximately 30% of the earth’s surface, Fe solubility is extremely low and does not match plant demand (Wallace and Lunt 1960; Marschner 1995). As a result, plants growing in such soils frequently suffer from Fe shortage, which causes poor chlorophyll production, manifesting as Fe deficiency chlorosis, lowering agricultural productivity and quality significantly. Fe uptake by plants is also a major source of Fe nutrition for animals and humans. The Fe concentration in cereal grains is very low, especially in rice. Thus, Fe deficiency anemia is a prevalent problem, affecting an estimated two billion people worldwide (WHO 2002), especially in Asian and African countries that rely on rice as a main cereal (Aung et al. 2013; Connorton and Balk 2019).

Plants have developed two specific mechanisms to acquire sparingly soluble Fe from the rhizosphere. Non-graminaceous plants utilize the reduction strategy (strategy I), in which ferrous Fe ions (Fe^2+^) are taken up by iron-regulated transporter (IRT) after reduction of Fe(III)-chelates by Fe deficiency-inducible ferric-chelate reductases (Marschner et al. 1986; Römheld and Marschner 1986). Graminaceous plants use the chelation strategy (strategy II), which is mediated by Fe(III) chelators, designated as mugineic acid family phytosiderophores (MAs) (Takagi 1976). Graminaceous plants produce 2’-deoxymugineic acid (DMA) and other MAs from *S*-adenosyl-L-methionine through a series of reactions catalyzed by nicotianamine synthase (NAS), nicotianamine aminotransferase (NAAT), deoxymugineic acid synthase (DMAS), and iron deficiency specific clone 2 (IDS2) and IDS3 dioxygenases (Shojima et al. 1990; Higuchi et al. 1999; Takahashi et al. 1999; Nakanishi et al. 2000; Kobayashi et al. 2001; Bashir et al. 2006). The synthesized MAs are secreted to the rhizosphere by transporter of mugineic acid family phytosiderophores 1 (TOM1) (Nozoye et al. 2011). In the rhizosphere, secreted MAs chelate ferric Fe, and the Fe(III)-MAs complexes are absorbed into the roots by Yellow Stripe 1 (YS1)/YSL transporters (Curie et al. 2001, 2009). Rice biosynthesizes DMA and utilizes Fe(III)-DMA as a strategy II mechanism, but this plant lacks IDS2 and IDS3 dioxygenases and is unable to synthesize further hydroxylated MAs (Nakanishi et al. 2000; Kobayashi et al. 2001). Instead, rice also takes up Fe^2+^ using OsIRT1 transporter as a partial strategy I mechanism (Ishimaru et al. 2006). Nevertheless, rice is highly susceptible to low Fe availability, due to the low capacity of DMA biosynthesis and the absence of Fe deficiency-inducible ferric-chelate reductase (Mori et al. 1991; Ishimaru et al. 2006). The ability of improved MA release under Fe insufficiency in graminaceous plants is strongly connected with their tolerance to Fe deficiency, for which barley and wheat outperform other common crops such as rice, maize, and sorghum (Römheld and Marschner 1986; Marschner 1995; Mori et al. 1991).

To date, several bioengineering approaches have enabled improvement in either Fe deficiency tolerance or Fe accumulation in rice by the introduction or manipulation of various genes involved in Fe uptake and transport. The most classical method to confer Fe deficiency tolerance on rice is the introduction of biosynthetic genes for MAs, typically from barley, such as *HvNAAT-A* and *-B*, *HvNAS1*, and *IDS3* (Higuchi et al. 2001; Kobayashi et al. 2001; Takahashi et al. 2001). Rice secretion of MAs was increased, and tolerance to low Fe availability was given in calcareous soil pots and even in fields after these genes were introduced with their own promoters (Kobayashi et al. 2001; Takahashi et al. 2001; Suzuki et al. 2008). This approach was generally effective in non-submerged conditions, but the tolerance was less evident in submerged conditions (Suzuki et al. 2008; Masuda et al. 2019). Another powerful method is the introduction of an artificial gene, *Refre1/372*, which is a mutationally reconstructed gene of the yeast ferric-chelate reductase gene *FRE1* (Dancis et al. 1990) to optimize codon usage for plants and to confer enhanced reductase activity even under high pH, that is, at around a pH of 9 (Oki et al. 2004). The introduction of *Refre1/372* downstream of the promoter of the rice Fe^2+^ transporter gene *OsIRT1* filled a gap in strategy I and conferred strong tolerance to low Fe availability in calcareous soils, particularly under submerged conditions (Ishimaru et al. 2007; Masuda et al. 2019). The third technique is increasing rice transcription factors that favorably regulate endogenous genes involved in Fe uptake. The introduction of iron deficiency-responsive element-binding factor 1 (*IDEF1*) gene, which positively regulates both strategy I and II genes (Kobayashi et al. 2007, 2009), under the control of the barley Fe deficiency-inducible *IDS2* promoter enhanced tolerance in calcareous soils at early stages (Kobayashi et al. 2007, 2013). Moreover, overexpression of rice iron-related transcription factor 2 (*OsIRO2*) gene, which positively regulates strategy II genes (Ogo et al. 2007), under the control of the constitutive *35S* promoter enhanced tolerance in calcareous soils throughout life stages (Ogo et al. 2011). Furthermore, combined introduction of multiple genes such as *OsIRT1* promoter-*Refre1/372* plus *35S* promoter-*OsIRO2* and further combination of barley *IDS3* resulted in superior tolerance in calcareous soils under various submerged and non-submerged conditions (Masuda et al. 2017, 2019).

Biotechnological approaches are also proven to be highly effective in the enhancement of Fe concentration in edible parts of rice, i.e. brown (hulled) seeds and polished seeds (endosperm) (reviewed in Bashir et al. 2013; Connorton and Balk 2019; Kawakami and Bhullar 2021). Because it does not necessitate the creation of specific infrastructure or drastic changes in existing habits, such biofortification is expected to be an excellent technique for preventing and treating global anemia. The pioneer method for rice biofortification is the ectopic expression of the Fe storage protein gene *Ferritin* in rice endosperm using endosperm-specific promoters (Goto et al. 1999; Qu et al. 2005). Another effective method is enhancement of Fe chelator nicotianamine (NA) by the introduction or overexpression of *NAS* genes (Masuda et al. 2009; Lee et al. 2009). NA is a chelator of divalent metals including Fe^2+^ for internal translocation (Curie et al. 2009). Also, it is a precursor of MAs, which are also responsible for internal Fe transport in addition to Fe uptake (Curie et al. 2009; Kobayashi et al. 2019a). Such Fe transport function of NA is also engineered to enhance Fe flow into endosperm by expression of the Fe(II)-NA transporter gene *OsYSL2* under the control of the sucrose transporter *OsSUT1* gene promoter (Ishimaru et al. 2010), which drives efficient expression in phloem cells and around the endosperm (Hirose et al. 2002). Introduction of the barley *IDS3* gene with its own promoter also moderately increases rice grain Fe concentrations (Masuda et al. 2008). Combining the above-mentioned genes such as *Ferritin, NAS*, *OsYSL2*, and *IDS3* further increases grain Fe accumulation by up to two-fold in brown rice and six-fold in polished rice (Wirth et al. 2009; Masuda et al. 2012; Aung et al. 2013; Masuda et al. 2013). Overexpression of the Fe transporter *OsIRT1* (Lee and An 2009) or the barley Fe(III)-MAs transporter *HvYS1* (Banakar et al. 2017) and disruption of tonoplast Fe transporter *OsVIT* (Zhang et al. 2012) or tonoplast DMA transporter *OsVMT/OsZIFL12* (Che et al. 2019) are two other gene manipulations that moderately increase Fe accumulation. In addition, the distribution of Fe within the rice grain could be modified by knockdown of *OsYSL9*, an Fe(III)-DMA and Fe(II)-NA transporter (Senoura et al. 2017). A recent report has shown that tissue-specific expression of *ZmYS1* and *TOM1* transporters downstream of *OsHMA2* and *OsFRDL1* promoters, respectively, is also effective in Fe accumulation in polished rice, especially when combined with introduction of *Ferritin* and *NAS* genes (Kawakami et al. 2022).

In many cases, such Fe-fortified rice grains also moderately accumulate zinc (Zn) (Masuda et al. 2008; Lee et al. 2009; Masuda et al. 2009; Wirth et al. 2009; Ogo et al. 2011; Masuda et al. 2012; Zhang et al. 2012; Aung et al. 2013; Masuda et al. 2013; Kawakami et al. 2022). This phenomenon could be explained by partly shared pathways of Fe and Zn translocation, including some transporters and common chelation by NA (Curie et al. 2009). This is also a useful property for biofortification, as Zn insufficiency is a serious nutritional problem that affects people all over the world (Wessells and Brown 2012). Reduced Fe antinutrient phytate, either through ectopic expression of a *Phytase* gene in the endosperm (Wirth et al. 2009) or reduction of phosphate accumulation in the endosperm due to knockout of a phosphate transporter gene, *SPDT* (Yamaji et al. 2017), has also been pursued to improve Fe bioavailability for human digestion in rice grains.

Despite the fact that numerous genes involved in Fe absorption and translocation are linked to both Fe deficiency tolerance and Fe accumulation in grains, the contribution rates to these two traits vary greatly depending on gene types. Irrespective of the importance of these traits, Fe deficiency-tolerant plants and Fe-fortified plants are generally analyzed separately; very limited information is present on the Fe accumulation traits of Fe deficiency-tolerant plants and *vice versa*. Such information includes Fe deficiency-tolerant *OsIRO2* overexpression rice lines, which also accumulated 2–3 times more Fe concentration in grains after pot cultivation in calcareous soils (Ogo et al. 2011). However, this increase in Fe concentration was not reproduced in another study, which reported increased Fe content in grain or straw per plant due to enhanced yield (Masuda et al. 2017), but no increase in Fe concentration *per se*. These reports did not analyze Fe accumulation further. Another report analyzed transgenic rice lines with introduced soybean *Ferritin* genes driven by two endosperm-specific promoters, along with barley biosynthetic genes for MAs, namely, *HvNAS1*, *HvNAAT-A* and *-B*, and *IDS3* genes with their own promoters (Masuda et al. 2013). These lines accumulated up to 4- and 2.5-times more Fe concentrations in polished seeds after cultivation in normal (Fe-sufficient) and calcareous soil pots, respectively, along with moderate but transient tolerance to Fe deficiency in the latter condition (Masuda et al. 2013). A field trial of a transgenic rice line with the barley *IDS3* gene showed Fe deficiency tolerance in calcareous soils (Suzuki et al. 2008) and moderate Fe accumulation in grains after Andosol soil cultivation (Masuda et al. 2008). None of these rice lines had strong traits for both Fe deficiency tolerance and Fe fortification in grains at the same time. Also, other bioengineered rice lines have been analyzed for only Fe deficiency tolerance or Fe fortification, but not for both. Concurrent improvement of these two features would be useful for future biofortified crop application in a variety of soil types as well as in fluctuating settings, wherein Fe deficiency can arise even in neutral or slightly acidic soils.

Previously, we have identified rice ubiquitin ligases, hemerythrin motif-containing RING- and Zn-finger protein 1 (*OsHRZ1*) and *OsHRZ2*, which negatively regulate Fe deficiency response and accumulation in rice (Kobayashi et al. 2013). *OsHRZ1* and *OsHRZ2* repress the expression of most of the known Fe deficiency-inducible genes for Fe uptake and translocation at the transcript level (Kobayashi et al. 2013), most likely through ubiqutination and subsequent degradation of some positive regulators, including bHLH and bZIP transcription factors and glutaredoxins (Zhang et al. 2017; Kobayashi et al. 2019b, 2022). Although knockout of *OsHRZ1* results in severe yield loss (Kobayashi et al. 2013), moderate knockdown of *OsHRZ1* and *OsHRZ2* confers substantial tolerance to Fe deficiency in calcareous soil pots, along with a 2‒3 time increase in Fe concentration in grains and leaves under both calcareous and Fe-sufficient soil cultivation, without apparent growth penalty (Kobayashi et al. 2013). These *OsHRZ* knockdown lines also accumulate approximately 1.5 times the Zn concentration in grains under both soil conditions (Kobayashi et al. 2013). Recently, IRON MAN (IMA)/Fe-uptake-inducing peptide (FEP) have been identified as potent inhibitors of the function of OsHRZs and BRUTUS (BTS)/BTS-like (Li et al. 2021; Lichtblau et al. 2022; Peng et al. 2022), which are functional homologs of OsHRZs in *Arabidopsis* (Long et al. 2010; Kobayashi et al. 2013; Hindt et al. 2017). In rice and *Arabidopsis*, the overexpression of IMA/FEPs causes Fe deficiency tolerance and a significant accumulation of Fe in the shoots (Grillet et al. 2018; Hirayama et al. 2018; Kobayashi et al. 2021; Peng et al. 2022). Although these phenotypes are similar to *HRZ/BTS* knockdown (Long et al. 2010; Kobayashi et al. 2013), *IMA/FEP*-overexpressing rice and *Arabidopsis* suffer from leaf bronzing or growth retardation, presumably due to Fe toxicity (Grillet et al. 2018; Kobayashi et al. 2021). In contrast, *OsHRZ* knockdown rice lines showed no obvious deleterious effects, with the exception of susceptibility to extreme Fe excess (Kobayashi et al. 2013; Aung et al. 2018). Thus, *OsHRZ* manipulation is a promising method for concomitant enhancement of Fe deficiency tolerance and Fe fortification. Because Fe deficiency tolerance of *OsHRZ* knockdown rice lines appear to be weaker than other lines with multiple gene introduction (Kobayashi et al. 2013; Masuda et al. 2017, 2019), there is a possibility for further enhancement of the former lines by additional bioengineering.

In the present report, we aimed to further enhance the Fe deficiency tolerance and grain Fe accumulation of *OsHRZ* knockdown rice by combining the introduction of the *OsHRZ* knockdown cassette with the *OsIRT1* promoter-*Refre1/372* cassette, the latter of which is known to confer strong Fe deficiency tolerance but not Fe accumulation (Ishimaru et al. 2007). Because the traits of *OsHRZ* knockdown rice relies on enhancement of endogenous gene expression for Fe uptake and translocation (Kobayashi et al. 2013), we hypothesized that further addition of exogeneous Fe deficiency-inducible ferric-chelate reductase activity which is absent in rice, by introduction of *OsIRT1* promoter-*Refre1/372*, could be complementary and highly effective. We obtained transgenic lines with altered levels of *OsHRZ* and *Refre1/372* expression, as well as Fe and Zn accumulation. Furthermore, the chosen lines demonstrated enhanced tolerance to low Fe availability in calcareous soil pots, sometimes outperforming single-introduced lines. These findings suggest that increasing endogenous Fe absorption and translocation in combination with artificially introducing ferric-chelate reductase is an efficient strategy to improve rice Fe deficiency tolerance and grain Fe fortification at the same time.

## Materials and methods

### Vector Development and Transgenic Rice Production

**Fig. 1:**
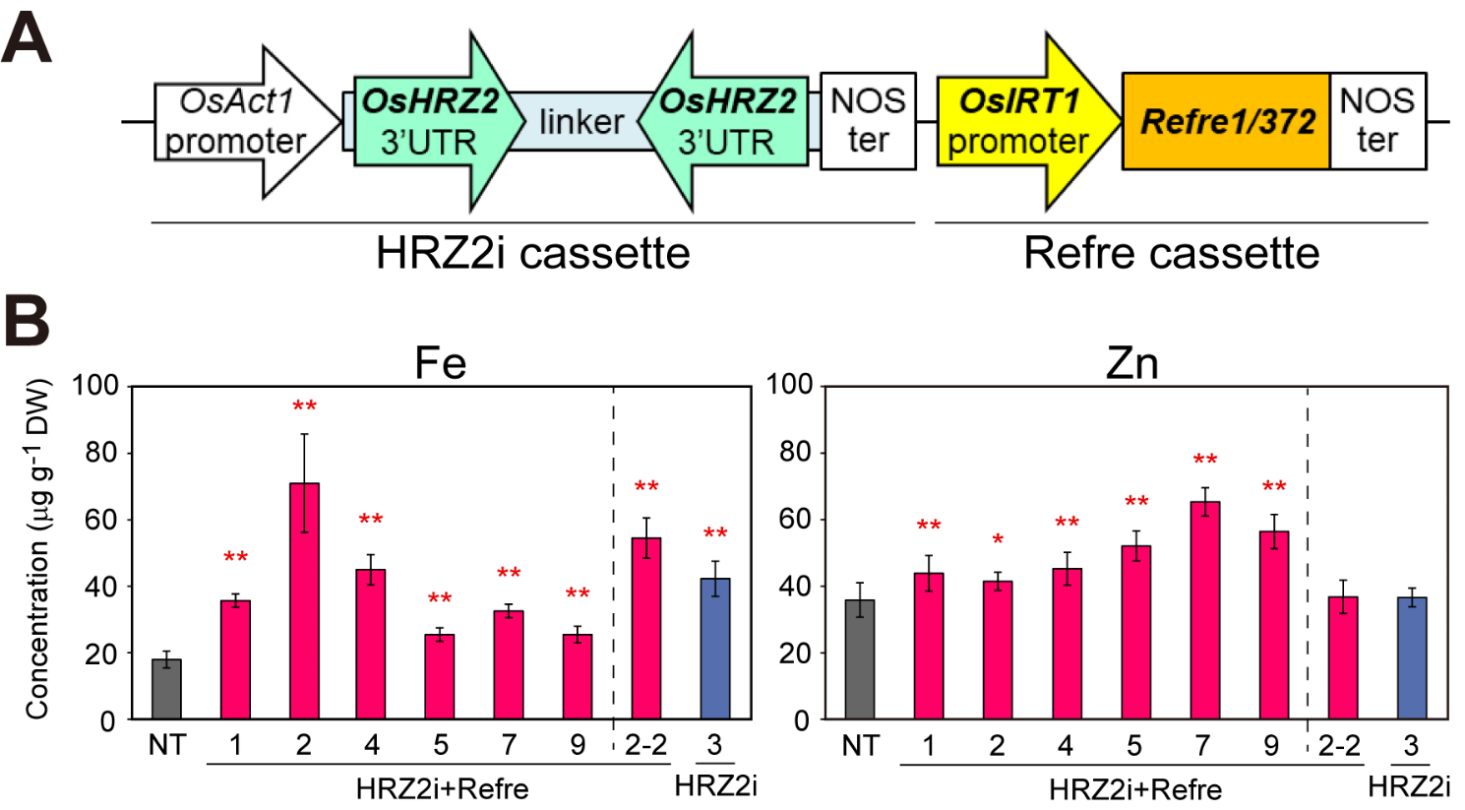
The vector structure and metal concentrations of the transgenic lines under Fe sufficiency. **A** HRZ2i + Refre transformant vector structure. The transformed binary vector contained the depicted HRZ2i and the Refre cassettes, along with hygromycin and kanamycin resistance genes inside the T-DNA region of the pIG121Hm vector backbone (Hiei et al. 1994). *OsAct1*, rice *Actin1* gene; 3’UTR, 3’ untranslated region; NOS ter, *Agrobacterium tumefaciens* nopaline synthase terminator. The vector is not drawn to scale. **B** Fe and Zn concentrations in the brown seeds after pot culture in Fe-sufficient soil. The following seeds were analyzed: NT, non-transformants; HRZ2i + Refre, T_1_ seeds of lines 1, 2, 4, 5, 7, and 9, and T_2_ seeds of line 2–2; HRZ2i, T_2_ seeds of line 3 (Kobayashi et al. 2013). Means and standard deviation (SD) are shown (n = 7 for NT, n = 6 for HRZ2i + Refre line 2 and 5; n = 5 for HRZ2i line 3; n = 4 for HRZ2i + Refre line 7 and 9; n = 3 for HRZ2i + Refre line 1, 4, and 2–2). DW: dry weight. Asterisks indicate significant differences compared to the NT (two-sample Student’s *t*-test; *P < 0.05; **P < 0.01)

We utilized In-Fusion cloning (TaKaRa, Japan) to incorporate the RNA interference (RNAi) cassette of the *OsHRZ2* 3’ untranslated region (3’UTR) fragment (HRZ2i cassette, Fig. 1 A) into the binary vector carrying the *Refre1/372* gene downstream of the *OsIRT1* promoter (Refre cassette, Fig. 1 A). For this purpose, two portions of the HRZ2i cassette were amplified by PCR using the *OsHRZ2*-RNAi vector (Kobayashi et al. 2013) as the template and were fused with the pBluescript SK (+) plasmid (Stratagene, CA, USA) amplified by PCR. The primers used are shown in Additional file 1: Table S1. After sequence verification, the HRZ2i cassette was excised using the PCR-introduced *Hin*dIII sites at both ends and inserted into the *Hin*dIII site of the binary vector carrying the Refre1 cassette (Ishimaru et al. 2007). The resultant binary vector containing both the HRZ2i and Refre1 cassettes (Fig. 1 A) was used for *Agrobacterium*-mediated transformation of rice (*Oryza sativa* L.) cultivar Tsukinohikari, as described previously (Hiei et al. 1994; Kobayashi et al. 2001). Regenerated plants (HRZ2i + Refre lines) were cultivated in Fe-sufficient normal soil as described below to obtain T_1_ seeds.

### Plant Materials and Growth Conditions

**Fig. 2:**
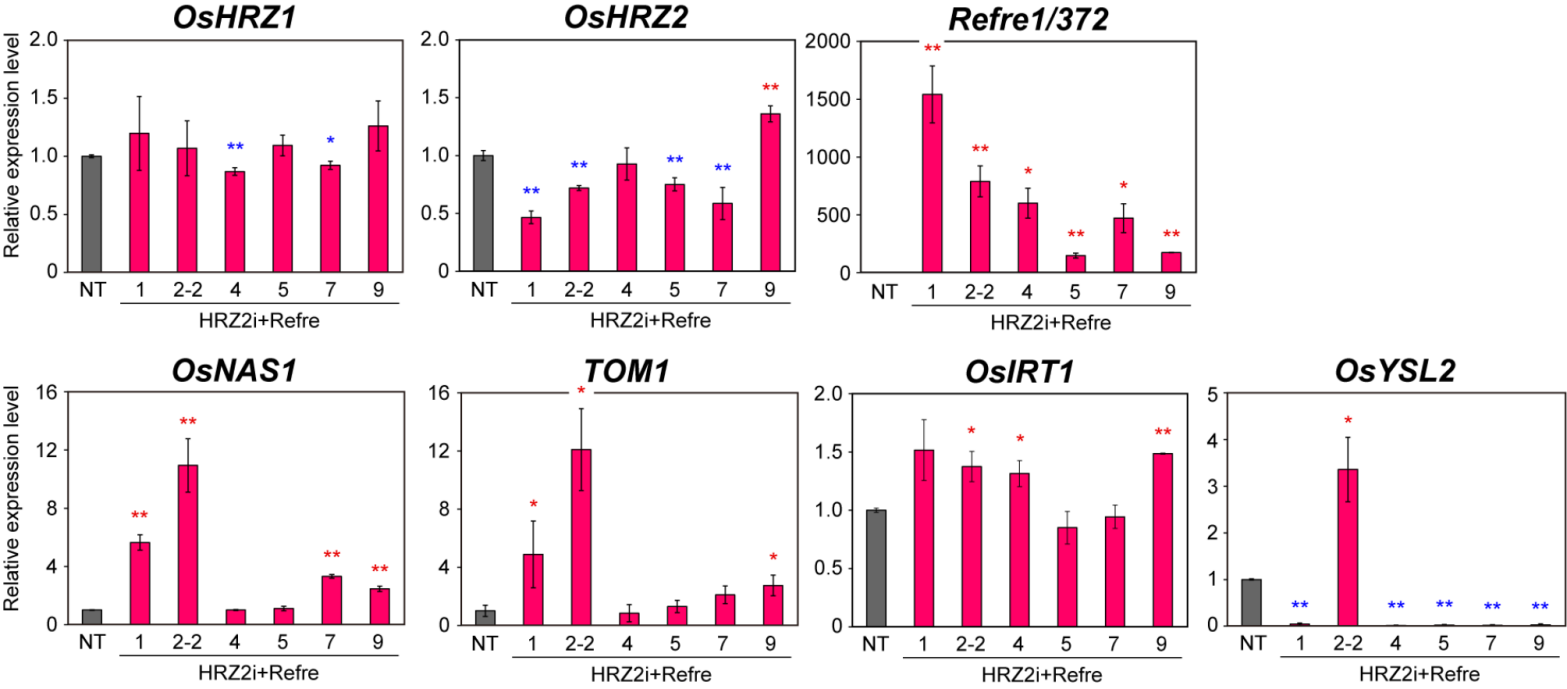
Expression levels of Fe-related genes in the roots of the HRZ2i + Refre lines. Transcript levels of *OsHRZ1*, *OsHRZ2*, *Refre1/372*, *OsNAS1*, *TOM1*, *OsIRT1* and *OsYSL2* were quantified by reverse transcription (RT)-PCR after Fe-sufficient hydroponic culture. Transcript abundance was normalized to rice *α-2 tubulin* and expressed as a ratio to the NT. The means ± SD are shown (n = 3). Asterisks indicate significant differences compared to the NT (two-sample Student’s *t*-test; *P < 0.05; **P < 0.01)

In addition to the above-mentioned HRZ2i + Refre lines, we used rice (cultivar Tsukinohikari) of non-transformants (NT), HRZ2i line 3 (T_1_ and T_2_ generations; Kobayashi et al. 2013), and Refre line 7 (T_3_ generation; Ishimaru et al. 2007). Germination, seedling culture and greenhouse cultivation in hydroponics and Fe-sufficient soil were carried out basically as described in Kobayashi et al. (2019b) as follows. Transgenic and NT seedlings were selected by germination with and without hygromycin B (50 mg L^− 1^), respectively, and each individual corresponded to each biological replicate. After acclimation period of 3–4 d, the seedlings (29-d old for Fe-sufficiency culture in Figs. 1B and 2, and 16-d old for Fe-deficiency and -sufficiency treatments in Additional file 1: Fig. S3) were transplanted to either hydroponic preculture or Fe-sufficient soil in a greenhouse under a 30 °C light/25°C dark cycle with natural light conditions. The composition of hydroponic solution was as described in Kobayashi et al. (2019b). Fe-sufficiency hydroponic culture in Fig. 2 was sustained for 8 d. Fe-deficiency and -sufficiency treatments in hydroponic culture in Fig. S3 were initiated after 5 d of preculture, and were sustained for 9 d. The Fe-sufficient soil was composed of a 1:1 mixture of Bonsol (Sumitomo Chemical, Japan) and vermiculite (Protoleaf, Japan) and supplied with 0.85 g each of EcoLongTotal 70 and LongTotal 140 controlled-release fertilizers (JCAM AGRI, Japan) in a 1-L pot.

**Fig. 3:**
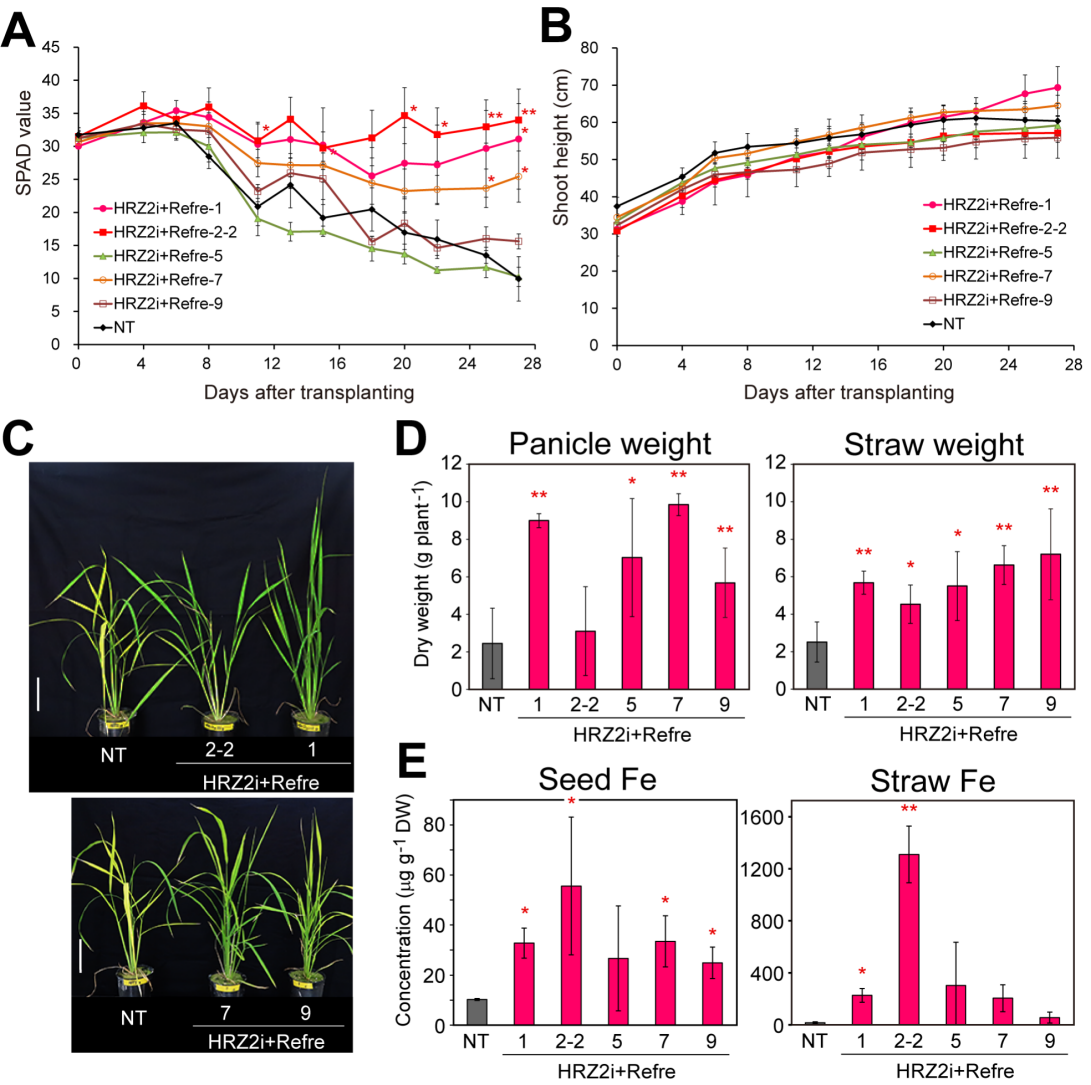
Features of the HRZ2i + Refre lines under pot cultivation in calcareous soil with non-submerged conditions. **A** The Soil Plant Analysis Development (SPAD) value of the newest leaves. **B** Shoot height. **C** Representative plants on day 29 after transplanting. Scale bar = 10 cm. **D** Panicle and straw dry weights after seed maturation. **E** Fe concentrations in brown seeds and straw following seed maturation. The following lines were cultivated: NT, non-transformants; HRZ2i + Refre, T_1_ lines of 1, 5, 7, and 9, and T_2_ line of 2–2. DW: dry weight. For **A** and **B**, the means ± standard error (SE) are shown (n = 4). Asterisks indicate significant differences compared to the NT at each time point (two-sample Student’s *t*-test; *P < 0.05; **P < 0.01). For **D** and **E**, the means ± SD are shown (n = 4 for all groups in **D** and HRZ2i + Refre lines 2–2, 5, 7, and 9 for seed Fe; n = 2 for NT for seed Fe; and n = 3 for HRZ2i + Refre line 1 for seed Fe and all lines for straw Fe). Asterisks indicate significant differences compared to the NT (two-sample Student’s *t*-test; *P < 0.05; **P < 0.01)

**Fig. 4:**
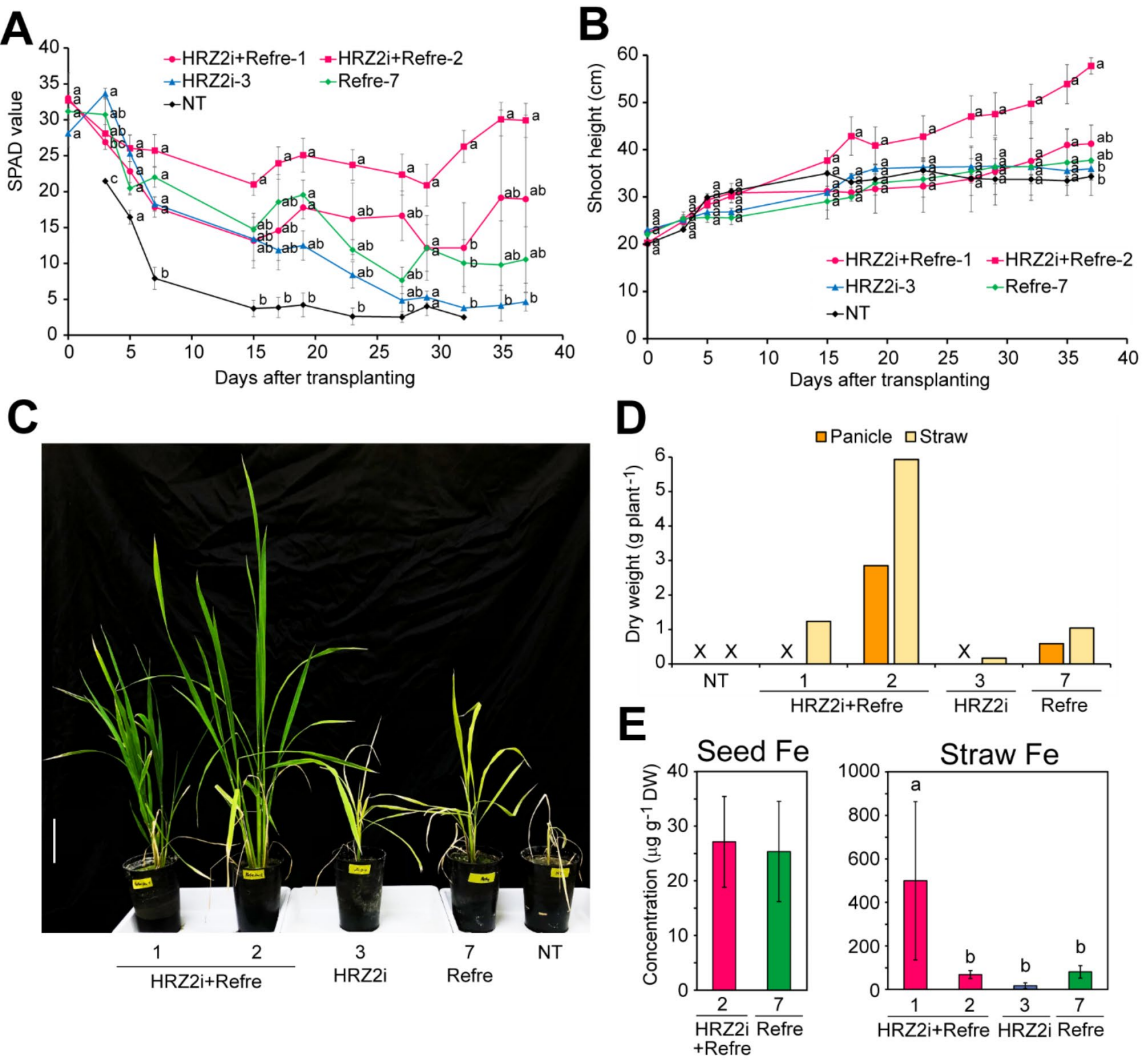
Features of the HRZ2i + Refre lines compared with single transformation lines in non-submerged calcareous soil. **A** SPAD value of the newest leaves. Data for NT on day 0 were around 30 but not recorded; data for NT on days 35 and 37 were unavailable due to the death of all new leaves. **B** Shoot height. **C** Representative plants on the 59th day after transplanting. Scale bar = 10 cm. **D** Panicle and straw dry weights after seed maturation. All the surviving plants were collected. An “X” mark indicates no yield. **E** Fe concentrations in the brown seeds and straw following seed maturation. The following lines were cultivated: NT, non-transformants; HRZ2i + Refre, T_1_ lines of 1 and 2; HRZ2i, T_2_ line 3; Refre, T_3_ line 7. DW: dry weight. For **A** and **B**, means ± SE are shown (n = 10 for HRZ2i + Refre line 2; n = 5 for other lines). For **E**, means ± SD are shown (n = 6 for HRZ2i + Refre line 2 for seed Fe; n = 3 for Refre line 7 for seed Fe; n = 7 for HRZ2i + Refre line 2 for straw Fe; n = 4 for other lines for straw Fe). Different letters indicate significant differences (*P* < 0.05; Tukey’s honest significant difference test). Datum for NT on day 32 was not used for this test due to the death of all new leaves except for one line

**Fig. 5:**
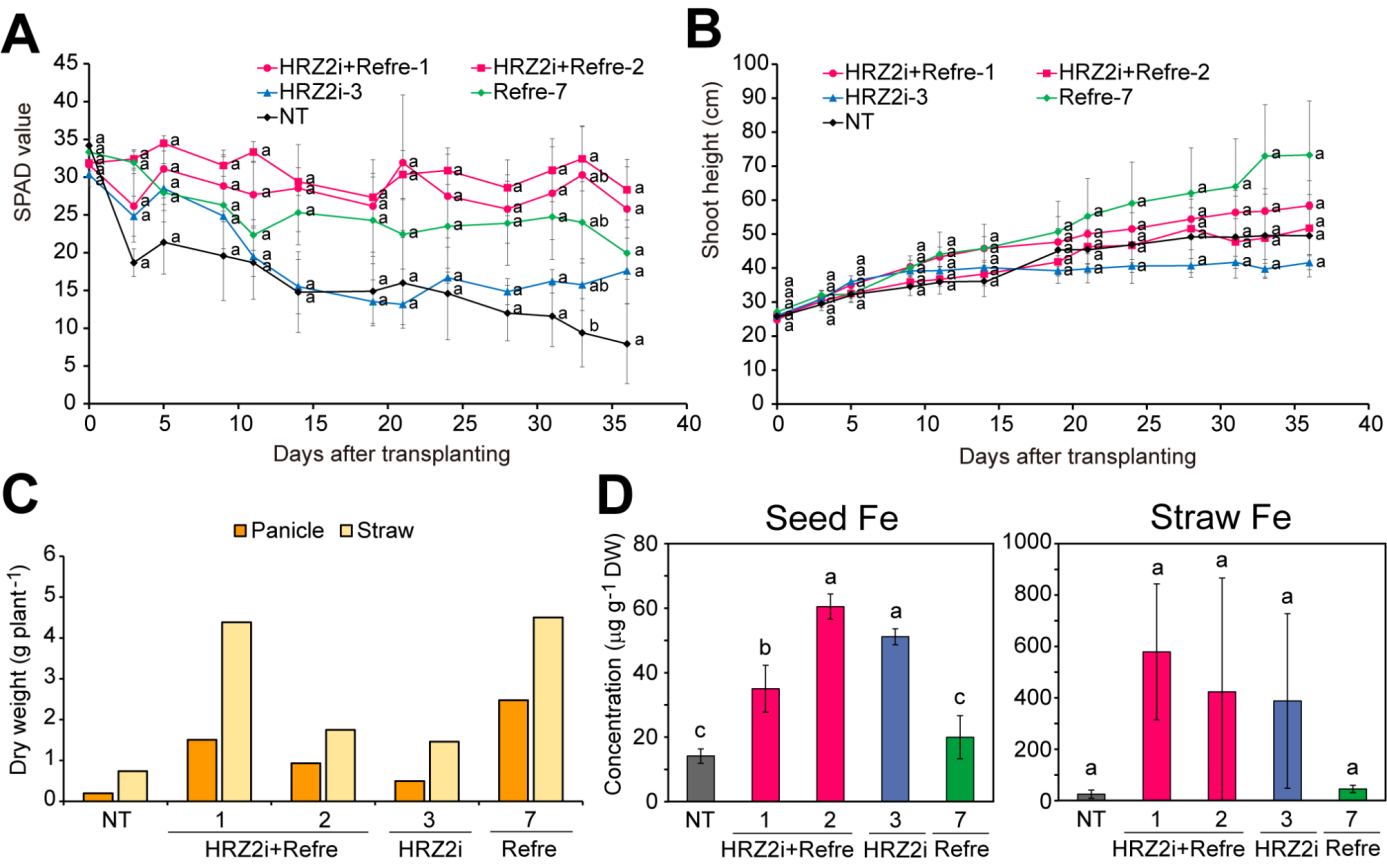
Features of the HRZ2i + Refre lines compared with single transformation lines in submerged calcareous soil. **A** SPAD value of the newest leaves. **B** Shoot height. **C** Dry weights of panicles and straw after seed maturation. All the survived plants were collected. **D** Fe concentrations in the brown seeds and straw following seed maturation. The following lines were cultivated: NT, non-transformants; HRZ2i + Refre, T_1_ lines of 1 and 2; HRZ2i, T_2_ line 3; Refre, T_3_ line 7. DW: dry weight. For **A** and **B**, the means ± SE are shown (n = 10 for HRZ2i + Refre line 2; n = 5 for other lines). For **D**, the means ± SD are shown (n = 3 for all lines for seed Fe and NT for straw Fe; n = 8 for HRZ2i + Refre line 2 for straw Fe; and n = 4 for other lines for straw Fe). Different letters indicate significant differences (*P* < 0.05; Tukey’s honest significant difference test)

Cultivation in calcareous soil pots was carried out in a greenhouse under a 30 °C light/25°C dark cycle with natural light conditions, as described in Kobayashi et al. (2013), with modifications as below. Seedlings after Fe-sufficient hydroponic preculture as described above were transplanted into a 1-L pot filled with autoclaved calcareous soil (pH 8.5–8.9) from Takaoka city, Toyama, Japan (Nihonkai Kougyou, Japan; Masuda et al. 2019) mixed with 0.85 g each of EcoLongTotal 70 and EcoLongTotal 140 (JCAM AGRI, Tokyo, Japan; Masuda et al. 2019). The pot contained holes at the bottom to supply water even under non-submerged conditions. The transplanting dates were as below: non-submerged cultivation in Fig. 3: 36 d (transformants) or 35 d (NT) after germination (7 d after the onset of preculture); non-submerged and submerged cultivations in Figs. 4 and 5: 21 d after germination (5 d after the onset of preculture). As below, tap water was supplied once every 2–3 d. Non-submerged condition in Figs. 3 and 4: up to half of the pot height; submerged condition in Fig. 5: up to a level slightly above the pot height. The Soil Plant Analysis Development (SPAD) value of the newest leaf and shoot heights were measured using an SPAD-502 chlorophyll meter (Konica Minolta, Tokyo, Japan). After seed maturation, the water supply was stopped, and plants and soil were air-dried before harvest.

### Metal Concentration Analysis

Nitric acid digestion and metal concentration analysis were carried out, as described in Kobayashi et al. (2013), with slight modifications as below. For each replicate, 6–10 brown seeds were dehusked and dried for 2 d at 70 °C, weighed, and wet-ashed with 1.0–1.5 mL of 13.4 M nitric acid and 1.0–1.5 mL of 8.8 M hydrogen peroxide for 20 min at 220 °C using a MarsXpress oven (CEM, Matthews, NC, USA). Straw segments were cut into 3–4 cm pieces and dried for 2–3 d at 70 °C, and portions weighing 100–200 mg were wet-ashed with 2.0 mL of 13.4 M nitric acid and 2.0 mL of 8.8 M hydrogen peroxide for 20 min at 230 °C. Inductively coupled plasma optical emission spectrometry (5800 ICP-OES, Agilent, Japan) was used to measure Fe, Zn, manganese (Mn) and copper (Cu) concentrations.

### Quantitative Reverse Transcription Polymerase Chain Reaction Analysis

Total RNA was extracted from the roots after Fe-sufficient hydroponic culture. RNA extraction, DNase treatment, reverse transcription, and quantitative PCR were performed, as described in Kobayashi et al. (2021), using the primers shown in Additional file 1: Table S1. After normalization with rice *α-2 tubulin* expression, transcript abundance was expressed as a ratio to the NT level.

### Statistical Analysis

For each set of comparisons, a two-sample Student’s *t*-test for equal or unequal variance was carried out based on an *F*-test for equal variance (significance level = 0.05) using Microsoft Excel software. Significant differences for multiple comparison were evaluated using Tukey’s honest significant difference test in the SPSS software (IBM, Tokyo, Japan).

## Results

### Transgenic Rice Lines with a Combination of ***OsHRZ*** Knockdown and ***Refre1/372*** Introduction (HRZ2i + Refre Lines) Accumulated Fe and Zn in Seeds

We constructed a binary vector carrying both the RNAi cassette of *OsHRZ2* 3’UTR fragment (HRZ2i cassette; Kobayashi et al. 2013) and the *Refre1/372* gene downstream of the *OsIRT1* promoter (Refre cassette; Ishimaru et al. 2007) (Fig. 1 A). Using *Agrobacterium*-mediated transformation, 38 transgenic lines were obtained, which we designated as HRZ2i + Refre lines. Among these, 35 lines set T_1_ seeds after cultivation in Fe-sufficient soil.

We examined the metal concentrations in the brown seeds of the representative HRZ2i + Refre lines (lines 1, 2, 4, 5, 7, and 9; Fig. 1B, Additional file 1: Fig. S1A). All these lines showed significantly higher concentrations of Fe and Zn than that of NT, with varying increased ratios against NT: ×1.4–4.0 for Fe and ×1.2–1.8 for Zn (Fig. 1B). Among these, line 2 showed the highest Fe accumulation. In Fe-sufficient soil, we obtained T_2_ seeds of this line (designated as line 2–2) and T_2_ seeds of previously produced HRZ2i line 3 (Kobayashi et al. 2013) for comparison. The T_2_ brown seeds of HRZ2i + Refre line 2–2 contained significantly more Fe concentration than that of NT, with an increased ratio of ×3.0, which tended to be slightly lower than the corresponding T_1_ seeds and higher than the T_2_ seeds of HRZ2i line 3 (×2.4) (Fig. 1B). These T_2_ seeds did not show an increased accumulation of Zn compared with that of NT (Fig. 1B). The Mn concentration in these T_1_ and T_2_ brown seeds varied greatly depending on the line, with many HRZ2i + Refre T_1_ lines having a moderately increased Cu concentration (Additional file 1: Fig. S1A).

We then analyzed the gene expression in the roots of these HRZ2i + Refre lines after Fe-sufficient hydroponic culture (Fig. 2), in which condition *HRZ* knockdown causes clearer alteration in downstream gene expression than in Fe-deficient condition (Kobayashi et al. 2013). Expression of *OsHRZ1* was slightly decreased in HRZ2i + Refre lines 4 and 7 but was unaltered in other lines, while expression of *OsHRZ2* was moderately decreased in HRZ2i + Refre lines 1, 2–2, 5, and 7 but unaltered in line 4 and increased in line 9. These results were generally in accordance with previous results of HRZ2i lines, which showed slight repression of *OsHRZ1* and moderate repression of *OsHRZ2* expression (Kobayashi et al. 2013). Expression of *Refre1/372* was strongly induced in all the HRZ2i + Refre lines to varying degrees, among which line 1 showed the strongest induction (Fig. 2). This confirms the successful introduction and expression of *Refre1/372* in these lines. We also examined four representative genes involved in Fe uptake and translocation: *OsNAS1*, *TOM1*, *OsIRT1* and *OsYSL2* (Fig. 2), of which its expression is enhanced in the HRZ2i lines under Fe sufficiency (Kobayashi et al. 2013). Among the HRZ2i + Refregenes, the expression of *OsNAS1* and *TOM1* was increased in lines 1, 2–2, 7, and 9 but not in lines 4 and 5. Line 2–2 showed the highest expression. Similar but weaker induction was also observed for *OsIRT1* expression, consistent with previous results of HRZ2i lines (Kobayashi et al. 2013). *OsYSL2* expression was strongly induced in line 2–2, but was strongly repressed in the other HRZ2i + Refre lines. Taken together, these results indicate the successful introduction and effect of either *HRZ* knockdown or *Refre1/372* in each HRZ2i + Refre line with varying degrees, among which line 1 showed the strongest *Refre1/372* expression, and line 2–2 showed the strongest de-repression of representative *HRZ* downstream genes.

### The HRZ2i + Refre Lines Show both Fe Deficiency Tolerance and Fe Accumulation Under Calcareous Soil Cultivation

We analyzed the growth features and metal accumulation of five HRZ2i + Refre lines (lines 1, 2–2, 5, 7, and 9) under pot cultivation in calcareous soil under non-submerged water management. We measured Fe nutritional condition and growth features by serial measurement of the SPAD value (relative chlorophyll concentration) of the newest leaf and shoot heights after transplanting to calcareous soil pots (Fig. 3 A, B). HRZ2i + Refre lines 1, 2–2, and 7 retained high SPAD values during the measurement period, but NT and HRZ2i + Refre lines 5 and 9 had clear chlorosis in new leaves after 8–11 d of transplanting (Fig. 3 A). The HRZ2i + Refre line 2–2 showed the highest SPAD values at many time points, which often showed significant differences against NT values. Lines 1 and 7 also showed significantly higher SPAD values compared with that of the NT after 27 d and some other time points (Fig. 3 A). All HRZ2i + Refre lines tended to have lower shoot heights at the start of treatment, although some lines tended to catch up to the NT height during treatment (Fig. 3B). At any timepoint, there was no significant variation in the shoot height between each line and the NT. After the measurement period, HRZ2i + Refre lines 2–2 and 1 clearly retained green leaves, while the NT leaves were severely chlorotic, and the HRZ2i + Refre lines 7 and 9 showed intermediate leaf colors (Fig. 3 C).

We harvested these plants after complete maturity of grains and measured the dry weights of panicles and straw (Fig. 3D). All the HRZ2i + Refre lines showed significantly higher panicle and straw weights than that of the NT, except for the panicle weight of line 2–2. Then, we measured the metal concentrations in the brown seeds and straw (Fig. 3E, Additional file 1: Fig. S1 B, C). All these HRZ2i + Refre lines accumulated much higher Fe concentrations than that of NT on average, although some lines did not show significant differences compared with that of NT because of high variations (Fig. 3E). Remarkably, the line 2–2 had extremely high Fe concentrations in both the brown seeds and straw (×5.4 and ×81 of NT, respectively), with significant differences when compared to that of the NT. Other lines showed increased ratios ×2.4–3.3 and ×3.4–19 of NT in the brown seeds and straw, respectively. These HRZ2i + Refre lines did not show marked alterations in Zn, Mn, or Cu concentrations compared with that of the NT in the brown seeds and straw (Additional file 1: Fig. S1B, C). These results indicate that some HRZ2i + Refre lines exhibit both Fe deficiency tolerance and Fe accumulation under calcareous soil cultivation.

### The HRZ2i + Refre Lines Show Similar or Better Growth and Fe Accumulation than that in Singly Introduced Lines under Non-submerged and Submerged Conditions in Calcareous Soil

We then compared the Fe deficiency tolerance and Fe accumulation of HRZ2i + Refre lines with previously characterized single introduction lines of either *HRZ* knockdown or *Refre1/372* introduction. For this purpose, we used HRZ2i + Refre lines 1 and 2, which showed typical and superior Fe deficiency tolerance and Fe accumulation (Figs. 1 and 3), along with HRZ2i line 3 and Refre line 7 as representative singly introduced lines, because the traits of these lines have been well characterized and shown to be typical and superior compared with other lines analyzed (Kobayashi et al. 2013; Ishimaru et al. 2007; Masuda et al. 2017, 2019). We used T_3_ generation for Refre line 7, which showed similar Fe deficiency tolerance at T_1_, T_2_ and T_3_ generations (Ishimaru et al. 2007; Masuda et al. 2017, 2019). Because the appearance of Fe deficiency phenotypes and the effect of each gene manipulation are much affected by growth conditions such as water management (Masuda et al. 2017, 2019), we used two water conditions for calcareous soil pot experiments: non-submerged condition (Fig. 4) and submerged condition (Fig. 5). For these experiments, the preculture lengths before transplanting were shortened compared to the previous experiment in Fig. 3 to impose a severer Fe deficiency.

Under such severer non-submerged conditions (Fig. 4), the NT seedlings rapidly showed chlorosis in the newest leaves as early as day 3 after transplanting, when HRZ2i + Refre line 2, HRZ2i line 3, and Refre line 7 showed significantly higher SPAD values than that in the NT (Fig. 4 A). The transgenic lines gradually became chlorotic to varying degrees but retained higher averaged SPAD values than that in the NT until day 23. Among these lines, HRZ2i + Refre line 2 retained the highest averaged SPAD values, with only a slight decrease from the start point and with significantly higher SPAD values than that in the NT at many time points. The SPAD values for HRZ2i + Refre line 1, HRZ2i line 3, and Refre line 7 remained moderate. Leaves of the NT died almost completely after day 32, while other lines survived. HRZ2i + Refre lines 1 and 2 showed moderate recovery of SPAD values after day 30 (Fig. 4 A).

The shoot height of the HRZ2i + Refre line 2 constantly increased during the experimental period, with significantly higher values than that in the NT on day 37 (Fig. 4B). The shoot height of HRZ2i + Refre line 1 also slowly increased throughout the experimental period but did not show a significant difference compared with that of the NT. The increase in the shoot height of HRZ2i line 3 and Refre line 7 was very low before day 15, but these lines retained growth thereafter. The increase in shoot height of NT almost stopped on day 15 (Fig. 4B).

After the measurement period, HRZ2i + Refre lines 1 and 2 further recovered from chlorosis, of which the green and big shoots became obviously superior to HRZ2i line 3 and Refre line 7, which suffered from severe chlorosis and stunted growth (Fig. 4 C). HRZ2i + Refre line 2 and Refre line 7 set matured seeds, but other lines did not (Fig. 4D). HRZ2i + Refre line 2 yielded the highest dry weight of both panicles and straw. HRZ2i + Refre line 1 and Refre line 7 produced moderate straw weight, whereas HRZ2i line 3 produced very little straw, but NT did not (Fig. 4D).

We measured the metal concentrations in the obtained brown seeds and straw (Fig. 4E). In the brown seeds, HRZ2i + Refre line 2 and Refre line 7 contained similar Fe concentrations. In the straw, HRZ2i + Refre line 1 contained a much higher Fe concentration than that in the other survived lines (Fig. 4E). The Zn, Mn, and Cu concentrations were roughly similar between the survived lines (Additional file 1: Fig. S2 A and B).

Another calcareous soil test was carried out under submerged conditions (Fig. 5). The NT seedlings showed chlorosis in the newest leaves as early as day 3 after transplanting, but the chlorosis did not progress as severe as that in the corresponding non-submerged condition (Fig. 5 A versus Fig. 4 A). Under submerged conditions, HRZ2i + Refre line 2 retained the highest SPAD values on avarage, with little decrease from the start point throughout the experimental period (Fig. 5 A). HRZ2i + Refre line 1 showed similar but slightly lower SPAD values than that in line 2. Refre line 7 retained moderate SPAD values, whereas HRZ2i line 3 showed little tolerance compared with that in the NT with regard to SPAD values under this condition (Fig. 5 A).

Under this condition, the shoot height was gradually increased during the experimental period in each line, but with no significant differences compared with that of the NT at any time point (Fig. 5B). During the late stages, Refre line 7 and HRZ2i line 3 showed the highest and lowest shoot height, respectively. All the lines yielded panicles and straw to varying degrees (Fig. 5 C). Refre line 7 and HRZ2i + Refre line 1 showed higher yields of panicles and straw than that of other lines.

In the brown seeds, HRZ2i + Refre lines 1 and 2, as well as HRZ2i line 3, had significantly higher Fe concentrations than in NT, with increased ratios of ×2.5, ×4.3, and ×3.6, respectively (Fig. 5D). These lines also had much higher Fe concentrations in straw than in NT, with increased ratios of ×23, ×17, and ×15, respectively, though a significant difference was not detected due to very high variations (Fig. 5D). In contrast, Refre line 7 showed only a slight and non-significant increase in the seed and straw Fe concentrations compared with that of the NT. The seed Mn concentration was lower in all the four lines compared with that in the NT, whereas the seed Cu concentration was higher in HRZ2i + Refre lines 1 and 2 compared with that in the NT (Additional file 1: Fig. S2C). The seed Zn concentration was higher in Refre line 7 compared with that in the NT (Additional file 1: Fig. S2C). The straw Zn, Mn, and Cu concentrations showed no clear alterations (Additional file 1: Fig. S2D).

We carried out additional Fe deficiency tolerance tests by hydroponic cultivation without Fe supply using HRZ2i + Refre line 2, HRZ2i line 3, Refre line 7, and NT (Additional file 1: Fig. S3). All the three transgenic lines showed significantly higher SPAD values of the newest leaves than that of NT at one or more time points (Additional file 1: Fig. S3A). HRZ2i line 3 showed the highest averaged SPAD values at each time point. After the 9-d treatment, HRZ2i + Refre line 2 and HRZ2i line 3 appeared greener than the other lines (Additional file 1: Fig. S3E). Shoot height was highest in Refre line 7, with significantly higher values than that in the NT on day 4 and thereafter (Additional file 1: Fig. S3B). Under Fe-sufficient conditions, all the lines retained SPAD values of around 30–35 during the treatment (Additional file 1: Fig. S3C). Shoot height was constantly increased under Fe sufficiency in all lines, among which Refre line 7 showed the highest values on average (Additional file 1: Fig. S3 D and F).

Overall, our findings showed that the HRZ2i + Refre, HRZ2i, and Refre lines were tolerant of low Fe availability under a variety of culture settings, with the degree of tolerance varying depending on genotype and culture circumstances. In all conditions evaluated, the HRZ2i + Refre lines showed stable Fe deficiency tolerance and Fe accumulation at stronger or comparable levels to that in singly introduced lines and NT.

## Discussion

Fe deficiency tolerance and Fe accumulation in edible parts are two major traits related to plant Fe nutrition. Recent reports have achieved enhancement of one of these two traits in crop species, especially rice, by the introduction or manipulation of various genes involved in Fe uptake, accumulation, and storage. Irrespective of the tight relationship between these two traits and related genes, little is known about concomitant enhancement of these two traits. In the present report, we took advantage of our previous achievement of *OsHRZ* knockdown rice lines, which show Fe deficiency tolerance under low Fe availability along with 2–3 times Fe accumulation in seeds and leaves irrespective of Fe nutritional conditions (Kobayashi et al. 2013; Aung et al. 2018). To further enhance these traits, we utilized combined introduction of *Refre1/372*, which is highly effective in conferring Fe deficiency tolerance when expressed under the *OsIRT1* promoter (Ishimaru et al. 2007; Masuda et al. 2019).

By transformation of the HRZ2i and Refre1 cassettes (Fig. 1 A), we successfully obtained rice lines with enhanced Fe and Zn concentrations in grains (Fig. 1B), as well as repressed expression of *OsHRZs* and introduced expression of *Refre1/372* (Fig. 2). These lines showed varying degrees of calcareous soil tolerance and Fe accumulation (Fig. 3). Among these lines, HRZ2i + Refre line 1 showed the highest expression of *Refre1/372* and strong repression of *OsHRZ2* (Fig. 2). Accordingly, this line demonstrated strong tolerance in calcareous soils until harvest, as well as highly increased Fe accumulation in seeds and straw (Fig. 3). HRZ2i + Refre line 2 was another notable line, with high tolerance in calcareous soils and the highest Fe accumulation in seeds and straw after calcareous soil cultivation (Fig. 3). This line also showed the highest Fe accumulation in seeds after normal soil cultivation (Fig. 1B) and the highest expression of *OsNAS1*, *TOM1*, and *OsYSL2* in Fe-sufficient roots (Fig. 2) among all the lines tested. *OsNAS1* and *TOM1* are important genes for MA-based Fe uptake and translocation (Inoue et al. 2003; Nozoye et al. 2011; Kobayashi et al. 2019a), whereas *OsYSL2* is involved in internal Fe(II)-NA and Mn(II)-NA translocation within the plant, particularly for grain Fe loading (Koike et al. 2004; Ishimaru et al. 2010). Expression of these genes is normally repressed under Fe sufficiency in NT rice but is de-repressed in the *OsHRZ* knockdown lines (Kobayashi et al. 2013). Curiously, *OsYSL2* expression was strongly enhanced in the HRZ2i + Refre line 2, similarly to previous *OsHRZ* knockdown lines, but was strongly repressed in the other HRZ2i + Refre lines (Fig. 2). In the latter lines, some secondary effects might have occurred for *OsYSL2* expression, possibly by affecting positive regulators of *OsYSL2* expression, such as IDEF1, IDEF2 and OsbZIP83 (Kobayashi et al. 2007, 2022; Ogo et al. 2008), or unknown negative regulators. Considering the increased Fe concentrations in all these HRZ2i + Refre lines (Fig. 1B), *OsYSL2* expression in these lines might be enhanced in shoot tissues critical for seed Fe loading, similarly to leaves of *OsHRZ* knockdown lines (Kobayashi et al. 2013). We also note that the expression of *Refre1/372* was very high in all the HRZ2i + Refre lines (Fig. 2) even though this gene was introduced under the control of the Fe deficiency-inducible *OsIRT1* promoter. This effect might be attributed to concomitant *OsHRZ* knockdown, because OsHRZs negatively regulate *OsIRT1* expression under Fe sufficiency (Kobayashi et al. 2013). Accordingly, expression of endogenous *OsIRT1* was also moderately induced in many HRZ2i + Refre lines (Fig. 2). Thus, the combined introduction of the HRZ2i cassette would be effective in the constitutive expression of *Refre1/372* in the Refre1 cassette, which might further potentiate the function of *Refre1/372* for Fe uptake. Thus, the strong Fe deficiency-tolerant and Fe-accumulating phenotypes of HRZ2i + Refre line 2 would be attributed to the strong de-repression of endogenous and exogenous Fe-related genes by the reduction of *OsHRZ* function.

We further indicated that the two selected lines, HRZ2i + Refre lines 1 and 2, exhibited stable Fe deficiency tolerance under both non-submerged and submerged cultivation in calcareous soils (Figs. 4 and 5), which was sometimes superior than that in single-introduced HRZ2i or Refre lines. In contrast, the tolerance of single-introduced lines was unstable and susceptible to water conditions. The Refre line 7 showed moderate tolerance under non-submerged conditions and better tolerance under submerged condition, in accordance with a previous report by Masuda et al. (2019), which suggested that submerged conditions are preferable to maintaining reduced Fe(II) generated by *Refre1/372* for subsequent uptake by OsIRT1 (strategy I). On the other hand, the HRZ2i line 3 showed moderate tolerance under non-submerged conditions and hydroponic culture (Fig. 4, Additional file 1: Fig. S3), similarly to our previous report (Kobayashi et al. 2013), but showed little tolerance under submerged conditions (Fig. 5). This could be owing to the latter condition’s dilution of increased MAs. Under submerged conditions, Fe deficiency tolerance of *OsIRO2* overexpression and barley *IDS3* introduction, both of which rely on expanded MAs (strategy II), has also been shown to be reduced (Masuda et al. 2019). Thus, concomitant enhancement of strategy II and strategy I contributes to extending the applicable conditions for enhanced Fe deficiency tolerance in rice (Masuda et al. 2019), which was further substantiated by the stable tolerance of our HRZ2i + Refre lines.

We confirmed Fe accumulation in the brown seeds and straw of the HRZ2i + Refre lines 1 and 2 after cultivation in calcareous soils (Fig. 3E). Early transplanting to non-submerged conditions resulted in the complete death of NT plants, precluding direct comparison of Fe concentration after harvest (Fig. 4). Submerged conditions resulted in the survival of all the lines, including NT, enabling direct comparison (Fig. 5). Under these conditions, the HRZ2i + Refre lines 1 and 2 showed a marked increase in Fe concentration in the seeds and straw compared to that in the NT, as did the HRZ2i line 3, but the Refre line 7 showed little increase (Fig. 5D). These results are in agreement with previous reports showing similar increases in Fe concentrations in seeds and straw of HRZ2i lines (Kobayashi et al. 2013) and only a slight increase in Fe concentration in shoots but not in seeds of Refre1 lines grown in calcareous soils (Ishimaru et al. 2007). HRZ2i + Refre line 2 and its progeny line 2–2, in particular, demonstrated stably high increase ratios in the Fe concentrations under various growth conditions: ×3.0–4.0 in Fe-sufficient soil (Fig. 1B), ×5.4 in non-submerged calcareous soil (Fig. 3E), and ×4.3 in submerged calcareous soil (Fig. 5D), which are comparable or superior to ×2–3.6 increase in HRZ2i lines (Figs. 1B and 5D; Kobayashi et al. 2013). These results indicate that addition of the Refre cassette into the *OsHRZ* knockdown contributes to further enhancement of Fe deficiency tolerance without compromising Fe accumulation, but rather extends the possibility of further increased Fe accumulation.

Fe toxicity is frequently caused by excessive Fe buildup in a given organ. However, we found no negative effects of Fe over-accumulation in the HRZ2i + Refre lines under our growing conditions. This is in contrast to *OsIMA*-overexpressing rice lines, which accumulate approximately × 2–4 increase in seed Fe concentration under Fe-sufficient soil cultivation but show severely retarded growth at early growth stages (Kobayashi et al. 2021). The *OsIMA*-overexpressing lines exhibit enhanced expression of most of the known Fe-related genes responsive to Fe deficiency, except *OsYSL2* (Kobayashi et al. 2021). In contrast, *OsYSL2* expression is strongly upregulated in *OsHRZ* knockdown lines (Kobayashi et al. 2013) and in the HRZ2i + Refre line 2 (Fig. 2). This feature might be a key to protecting cells from Fe toxicity even under high concentrations, possibly in combination with enhanced production of NA by the upregulation of *NAS*, the latter of which is known to confer Fe excess tolerance (Curie et al. 2009; Aung et al. 2019). This feature might also contribute to outstanding Fe deficiency tolerance in the HRZ2i + Refre line 2 (Figs. 3, 4 and 5). In addition to its protective role under Fe excess and enhanced tolerance to Fe deficiency, NA also serves as an important chelator for other divalent metals such as Zn and Cu (Curie et al. 2009). In our growth conditions, Zn and Cu concentrations in the seeds increased in many HRZ2i + Refre lines after Fe-sufficient soil cultivation (Fig. 1B, Additional file 1: Fig. S1A), but only Cu concentration increased after calcareous soil cultivation (Additional file 1: Fig. S1B, S2A and C). Soil types and water conditions might affect the uptake and accumulation rates of each metal depending on metal species. NA-based Fe accumulation is also thought to be preferable for human diets because *OsNAS3*-overexressing NA- and Fe-fortified rice seeds are effective in alleviating mouse anemia (Lee et al. 2009). Also, Fe(II)-NA is taken up in the small intestine *via* human transporter PAT1 (Murata et al. 2021) and improves Fe status in chickens (Beasley et al. 2020). Furthermore, NA overexpression is suggested to confer drought tolerance in rice (Lee et al. 2017). This is a highly promising trait for Fe deficiency-tolerant crops because calcareous soils are vastly distributed in arid and semi-arid regions worldwide. Because of the strong induction of the *NAS* and *OsYSL2* genes in the HRZ2i + Refre and HRZi lines (Fig. 2; Kobayashi et al. 2013), Fe(II)-NA and NA are expected to accumulate in these lines, potentially conferring the aforementioned beneficial traits such as improved Fe intake efficiency by humans and drought tolerance.

To date, various genes have been exploited to fortify Fe in edible parts of rice and other crops. Usually, multiple gene combinations are needed to fulfill high Fe accumulations (Masuda et al. 2012; Bashir et al. 2013; Kawakami and Bhullar 2021; Kawakami et al. 2022). Our *OsHRZ* knockdown method is highly advantageous over these methods in that it requires only one gene manipulation, mitigating the effort and labor of line selection and maintenance. This approach is also applicable to other methodologies such as conventional breeding and genome editing of *OsHRZs*. Further characterization of related genes and transformants will pave the way to the practical usage of *HRZ* mutations and modifications in various crop species.

## Conclusion

We produced transgenic rice lines combining *OsHRZ* knockdown with the *OsIRT1* promoter-*Refre1/372* introduction, which were conferred with increased tolerance to low Fe availability under various conditions in calcareous soils, as well as stably high Fe accumulations in seeds and straw.

## Electronic Supplementary Material

Below is the link to the electronic supplementary material.

Additional file 1: Table S1. Primers used in the present study. Fig. S1. Additional metal concentrations in the HRZ2i+Refre lines. Fig. S2. Additional metal concentrations in the HRZ2i+Refre lines compared with single transformation lines after pot cultivation in calcareous soil. Fig. S3. Features of the HRZ2i+Refre lines compared with single transformation lines under hydroponic culture.

## Declarations

### Data availability

The datasets used and/or analyzed during the current study are available from the corresponding author on reasonable request.

## Abbreviations

BTS: BRUTUS
DMA: 2’-deoxymugineic acid
HRZ: hemerythrin motif-containing RING- and Zn-finger protein
HRZ2i: the RNA interference cassette of the *OsHRZ2* gene
IDEF,: iron deficiency-responsive element-binding factor
IDS: iron deficiency specific clone
IMA/FEP: IRON MAN/Feuptake-inducing peptide
IRO2: iron-related transcription factor 2
IRT: iron-regulated transporter
MAs: mugineic acid family phytosiderophores
NA: nicotianamine
NAAT: nicotianamine aminotransferase
NAS: nicotianamine synthase
Refre: the *OsIRT1* promoter-*Refre1/372* gene cassette
Refre1/372: mutationally reconstructed variant 372 of the yeast ferric-chelate reductase 1
RNAi: RNA interference
TOM1: transporter of mugineic acid family phytosiderophores 1
UTR: untranslated region
YS1: Yellow Stripe 1
YSL: Yellow Stripe-like
