# Supplementary material for: Simultaneous Enhancement of iron Deficiency Tolerance and Iron Accumulation in Rice by Combining the Knockdown of OsHRZ Ubiquitin Ligases with the Introduction of Engineered Ferric-chelate Reductase
Source: Rice (N Y). 2022 Oct 31;15:54. doi: 10.1186/s12284-022-00598-w (PMC9622965; doi:10.1186/s12284-022-00598-w)
Supplement: Supplementary file 1 — Additional file 1: Table S1. Primers used in the present study. Fig. S1. Additional metal concentrations in the HRZ2i+Refre lines. Fig. S2. Additional metal concentrations in the HRZ2i+Refre lines compared with single transformation lines after pot cultivation in calcareous soil. Fig. S3. Features of the HRZ2i+Refre lines compared with single transformation lines under hydroponic culture. [file 12284_2022_598_MOESM1_ESM.pdf]

Additional file 1

Table S1. Primers used in the present study.

	Primer name	Primer sequence
Vector construction (underlined: extra sequence for subcloning)		
	HRZ2i cassette 5'-half Forward	TTCGATATC AAGCTT CGTATGTTGTGTGGAATTGTG
	HRZ2i cassette 5'-half Reverse	TTCAGTTCGTTGTTCACACAAAC
	HRZ2i cassette 3'-half Forward	GAACAACGAACTGAACTGGC
	HRZ2i cassette 3'-half Reverse	GGATTCGAT AAGCTT CCTAGAAGCTAATTCCCGATC
	pBluescript Forward	AAGCTT ATCGATACCGTCGACC
	pBluescript Reverse	AAGCTT GATATCGAATTCCTGC
RT-qPCR		
	OsHRZ1 RING-finger Forward	GAATTC CACAAATGCCGGGAGAAAGG
	OsHRZ1 RING-finger Reverse	AGCCAGCAAGGCGTCCAA
	OsHRZ2 RING-finger TaqMan	TaqMan Gene Expression Assays Os03496741_g1
	Refre1/372 Forward	CCGAGAAGGTCTTCAGGAAC
	Refre1/372 Reverse	CATCCATCCTAGTGTGTGGC
	OsNAS1 TaqMan	TaqMan Gene Expression Assays Os03604947_s1
	TOM1 Forward	CACCAGTTGCAGATCGTATAGGGAGGAA
	TOM1 Reverse	TCGGAAAATACATTTGGATATTGCT
	OsIRT1 Forward	CGTCTTCTTCTTCTCCACCACGAC
	OsIRT1 Reverse	GCAGCTGATGATCGAGTCTGACC
	OsYSL2 TaqMan	TaqMan Gene Expression Assays Os03560292_m1
	alpha-2 tubulin TaqMan	TaqMan Gene Expression Assays Os03562997_mH

Fig. S1

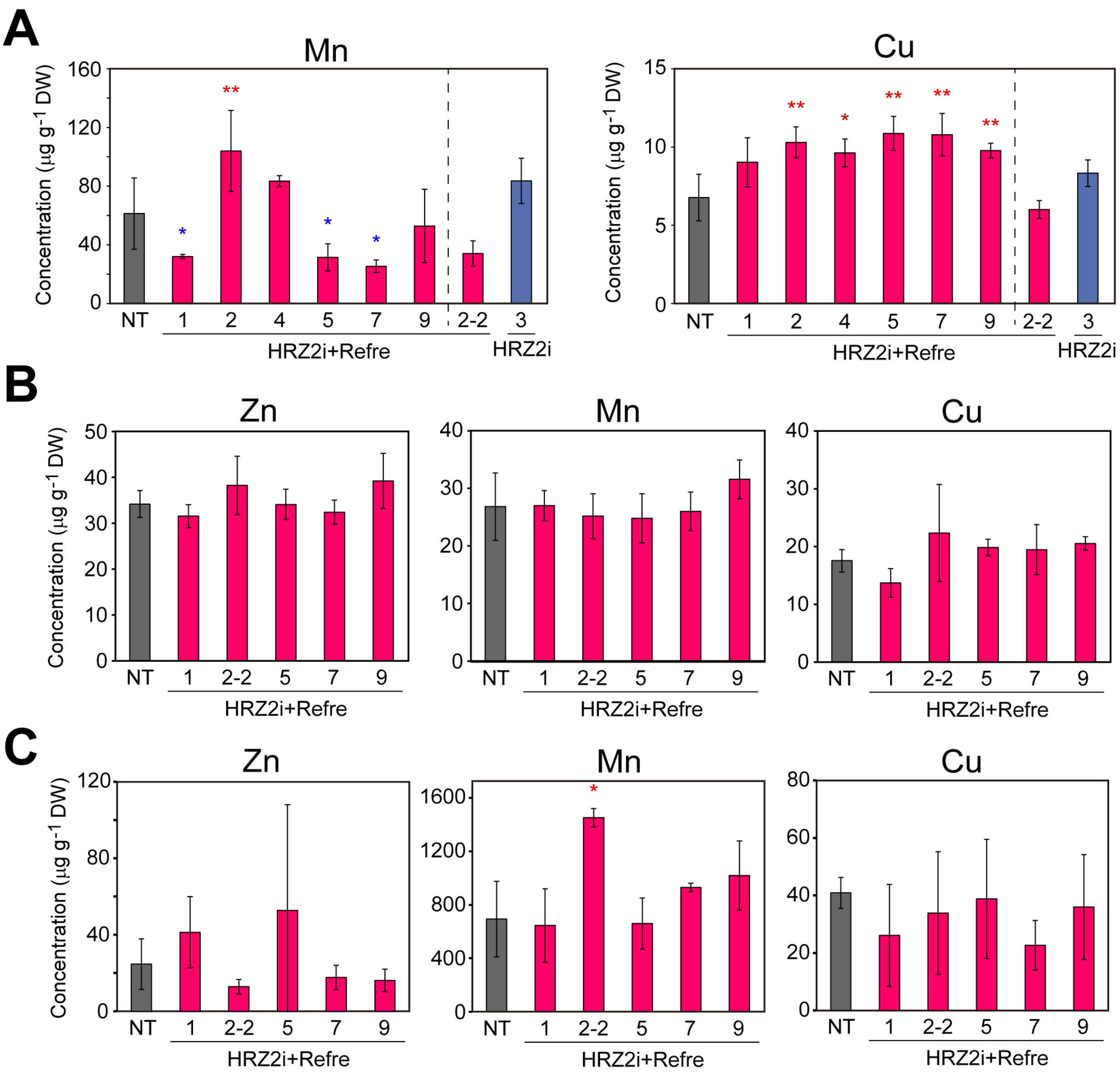

Fig. S1. Additional metal concentrations in the HRZ2i+Refre lines. **A** Mn and Cu concentrations in the brown seeds after pot culture in Fe-sufficient soil in Fig. 1B. The following seeds were analyzed: NT, non-transformants; HRZ2i+Refre, T_1 seeds of lines 1, 2, 4, 5, 7 and 9, and T_2 seeds of line 2-2; HRZ2i, T_2 seeds of line 3 (Kobayashi et al., 2013). Means ± SD are shown (n = 7 for NT, n = 6 for HRZ2i+Refre lines 2 and 5; n = 5 for HRZ2i line 3; n = 4 for HRZ2i+Refre lines 7 and 9; and n = 3 for HRZ2i+Refre lines 1, 4 and 2-2). **B, C** Zn, Mn, and Cu concentrations in the brown seeds (**B**) or straw (**C**) after pot cultivation and seed maturation in calcareous soil with non-submerged conditions in Fig. 3. The following lines were cultivated: NT, non-transformants; HRZ2i+Refre, T_1 lines of 1, 5, 7, and 9, and T_2 line of 2-2. The means ± SD are shown (n = 4 for HRZ2i+Refre lines 2-2, 5, 7, and 9 in **B**; n = 2 for NT in **B**; n = 3 for HRZ2i+Refre line 1 in **B** and all lines in **C**). DW: dry weight. Asterisks indicate significant differences compared to the NT (two-sample Student's *t*-test; $*P < 0.05$; $**P < 0.01$).

Fig. S2

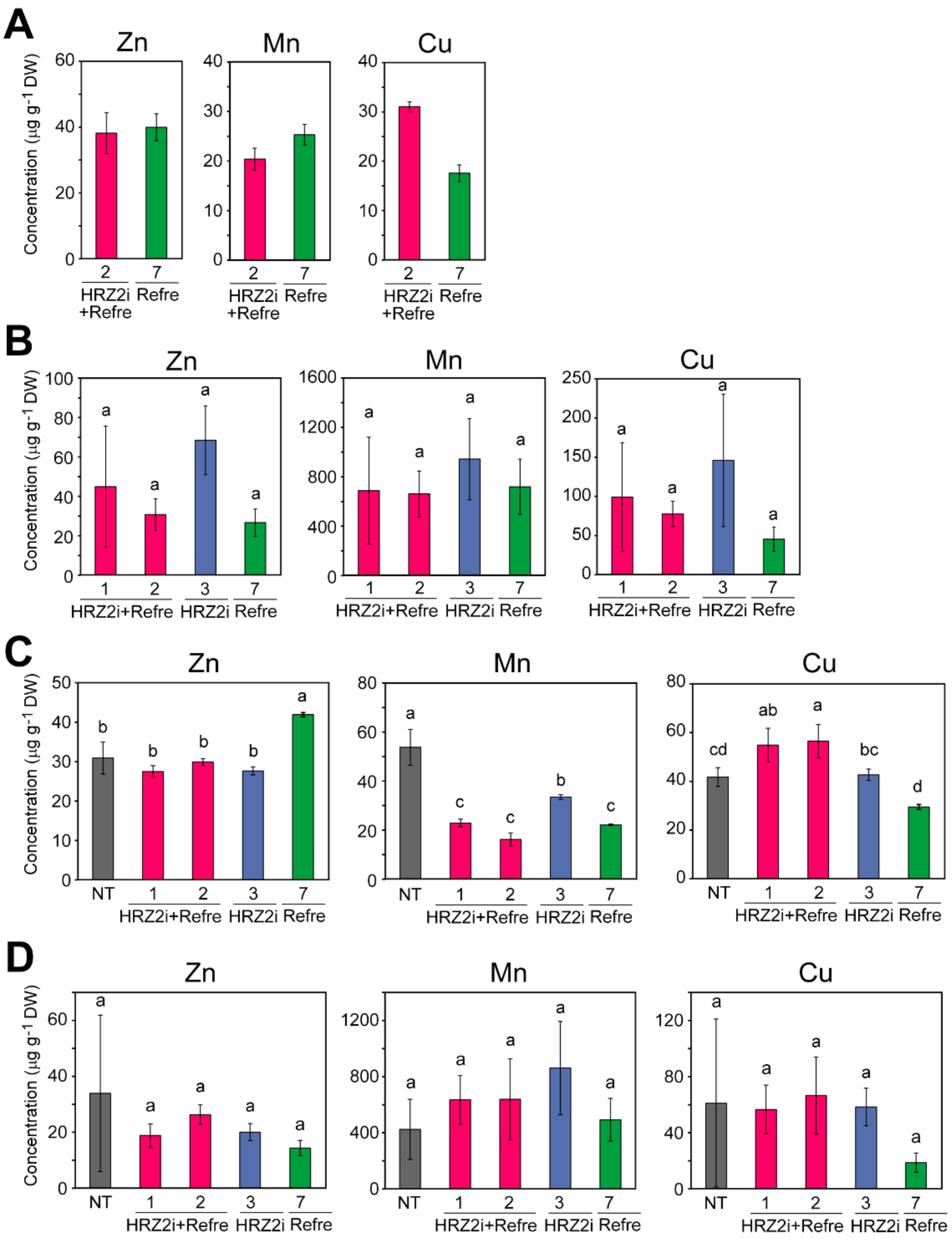

Fig. S2. Additional metal concentrations in the HRZ2i+Refre lines compared with single transformation lines after pot cultivation in calcareous soil. **A, B** Zn, Mn, and Cu concentrations in brown seeds (**A**) or straw (**B**) in the non-submerged conditions in Fig. 4. **C, D** Zn, Mn, and Cu concentrations in brown seeds (**C**) or straw (**D**) in the submerged conditions in Fig. 5. The following lines were cultivated: NT, non-transformants; HRZ2i+Refre, T_1 lines of 1 and 2; HRZ2i, T_2 line 3; Refre, T_3 line 7. The means ± SD are shown. **A, B**: n = 6 for HRZ2i+Refre line 2 for seed; n = 3 for Refre line 7 for seed; n = 7 for HRZ2i+Refre line 2 for straw; n = 4 for other lines for straw. **C, D**: n = 3 for all lines for seed and NT for straw; n = 8 for HRZ2i+Refre line 2 for straw; n = 4 for other lines for straw. DW: dry weight. Different letters indicate significant differences ($P < 0.05$; Tukey's honest significant difference test).

Fig. S3

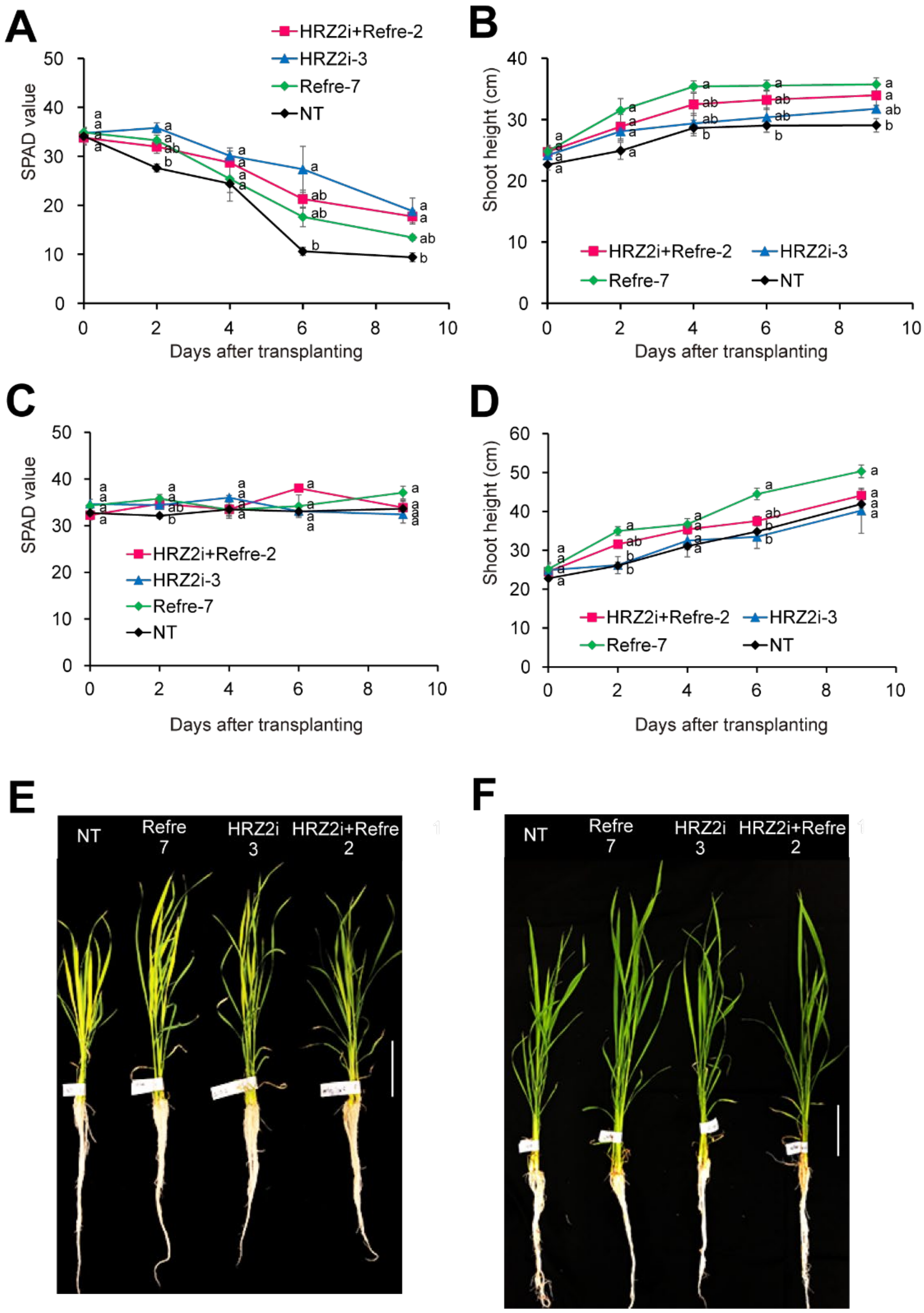

Fig. S3. Features of the HRZ2i+Refre lines compared with single transformation lines under hydroponic culture. **A** SPAD value of the newest leaves during Fe-deficient culture. **B** Shoot height during Fe-deficient culture. **C** SPAD value of the newest leaves grown in Fe-sufficient conditions. **D** Shooting height in Fe-sufficient culture. **E, F** Representative plants after 9 d of Fe-deficient (**E**) or Fe-sufficient (**F**) culture. Three plants were bundled. Scale bar = 10 cm. For **A-D**, the means ± SD are shown are shown (n = 3 HRZ2i line 3 in **C** and **D**; n = 4 for others). Different letters indicate significant differences ($P < 0.05$; Tukey's honest significant difference test).
